# Polymer-Based Coatings for Cardiovascular and Endovascular Devices: Linking Surface Chemistry, Drug Release Kinetics, and Thrombo-Inflammatory Performance: A Review

**DOI:** 10.3390/polym18121539

**Published:** 2026-06-20

**Authors:** Rasit Dinc, Nurittin Ardic

**Affiliations:** 1 Invamed Medical Innovation Institute, One World Trade Centre, 85th Floor, 285 Fulton Street, New York, NY 10007, USA; 2 Med-International UK Health Agency Ltd., Warwickshire, Nuneaton CV11 6LT, UK; nurittinardic@yahoo.com

**Keywords:** cardiovascular devices, polymer coatings, drug-eluting stents, surface chemistry, drug release kinetics, hemocompatibility, thrombo-inflammation

## Abstract

Polymer coatings are integral to nearly every modern cardiovascular and endovascular device, including drug-eluting stents (DESs) and drug-coated balloons (DCBs), bioabsorbable vascular scaffolds (BVSs), occluders, grafts, and catheter and guidewire hydrophilic surfaces. Persistent complications, including late stent thrombosis, delayed endothelialization, hypersensitivity, and restenosis, show that coatings actively shape biological responses rather than acting as inert drug carriers. Their surface chemistry, drug release kinetics, and degradation behavior are upstream determinants of blood– and tissue–material responses that govern healing and failure. This review frames coating selection as a structure–property–biological response problem. It surveys the major classes of synthetic polymer coatings and the defining surface and bulk properties. This review also examines how composition and architecture control drug release, and traces the interfacial cascade of protein adsorption, coagulation and complement activation, platelet and leukocyte responses, and neutrophil extracellular trap (NET) formation. These mechanisms are linked to contemporary design strategies that improve hemocompatibility, limit thrombosis, promote endothelial recovery, and tune degradation, and to the standardization and translation gaps that remain. The central message is that polymer coatings are not biologically equivalent. Their surface chemistries and degradation profiles determine the thrombo-inflammatory outcomes. Therefore, coating design should be guided by intended biological response, not drug release alone.

## 1. Introduction

Endovascular and cardiovascular device therapy has transformed the treatment of stenotic, thrombotic, and aneurysmal diseases, and polymer coatings are central to this transformation [1]. Drug-eluting stents (DESs) rely on thin polymer films to deposit and release antiproliferative drugs at controlled rates. Drug-coated balloons (DCBs) utilize polymer or excipient carriers to deliver the drug to the vessel wall during short-term inflation. Bioabsorbable vascular scaffolds (BVSs) are largely constructed from degradable polymers. Additionally, a wide variety of catheters, guidewires, grafts, occluders, and embolization systems rely on polymer surfaces for lubrication, hemocompatibility, or therapeutic function [2,3]. In this range of devices, the coating is the key interface between an engineered material and flowing blood and damaged tissue. This review focuses specifically on synthetic polymer coatings applied to blood-contacting cardiovascular and endovascular devices, rather than on structural device polymers or nonvascular applications. These coatings include drug-carrying and surface-modifying layers on stents, balloons, scaffolds, grafts, and catheters. Their principal classes are introduced in Section 2.

Despite thirty years of improvement, the clinical performance of coated devices remains limited by biological complications that drug optimization alone cannot resolve [4]. Pathology and preclinical studies of DESs have repeatedly associated them with delayed arterial healing, incomplete endothelialization, persistent fibrin deposition and inflammatory infiltration, late and very late stent thrombosis, and neoatherosclerosis [5,6]. Restenosis rates of approximately 5–20% in coronary DESs, and significantly higher rates in complex peripheral interventions, continue to trigger re-intervention [4,7]. More importantly, the same device can produce different outcomes in different patients [6,8]. This implies that healing trajectories are shaped by biological factors that interact with and are partly determined by the coating material itself.

The proximate causes of these complications are increasingly understood as thrombo-inflammatory: the intertwining of innate immunity and coagulation at the blood–material interface can be resolved toward healing or amplified toward thrombosis [9,10]. Coating influences each stage of this process. Their surface chemistry determines which plasma proteins will be adsorbed in which conformation, while release kinetics shape the local pharmacological and inflammatory environment over time. Furthermore, the degradation products of biodegradable coatings generate their own inflammatory stimuli. For example, recent studies have shown that neutrophil extracellular traps (NET) formation modulates the biocompatibility, degradation kinetics, and performance of biodegradable vascular scaffolds [11].

This review is deliberately positioned at the level of coating materials. This is not a device selection framework or a study toward AI-assisted monitoring or immune system-adaptive device design. These topics have been addressed elsewhere [12]. These studies are provided here as background information rather than being redeveloped. Instead, we treat the polymer coating as a moderating variable and ask how its structure and properties are reflected in the biological response. Section 2 examines coating classes and surface chemistry; Section 3 relates composition and architecture to drug release kinetics; Section 4 examines protein adsorption and hemocompatibility at the interface; Section 5 develops the thrombo-inflammatory response as a central determinant of outcome; Section 6 maps material design strategies to these pathways; and Section 7 addresses standardization and translation gaps. As a narrative review, this study integrates primary quantitative findings from heterogenous studies into a unifying structure–property–response framework, rather than reporting a single dataset.

## 2. Polymer Coating Classes and Surface Chemistry

Polymer coatings used in blood-contact devices fall into three broad functional categories that are distinguished from each other in terms of both surface physics and bulk composition: durable carriers, biodegradable carriers, and surface modifier or bioactive layers [13].

### 2.1. Durable Polymer Carriers

Durable carriers remain on the device for the entire functional life of the device and are valuable in terms of film integrity, drug biocompatibility, and reproducible release [14]. However, their relative thrombogenicity varies significantly depending on the chemistry. These observations demonstrate that polymer chemistry is a significant determinant of hemocompatibility and thrombotic risk [15,16].

Fluoropolymers, primarily poly(vinylidene fluoride-co-hexafluoropropylene) (PVDF-HFP), are semi-crystalline materials with a dense fluorinated carbon backbone. Their high carbon-fluorine bond dissociation energy makes them chemically inert and resistant to hydrolytic, oxidative, and enzymatic degradation, while their pronounced hydrophobicity determines how plasma proteins interact with the surface [15]. Comparative hemocompatibility tests have shown that PVDF-HFP exhibits a distinct protein adsorption pattern, preferring to retain albumin over fibrinogen. This pattern is thought to underlie the low thrombogenicity (“fluoropassivation”) of fluoropolymer-coated everolimus-releasing stents [16]. This material-level behavior helps explain the favorable clinical thrombosis profile of current fluoropolymer DESs [6].

From a coating design perspective, the importance of fluoropolymers lies not only in their chemical inertness but also in how this inertness translates to a specific blood-contact phenotype. Fluorinated surfaces exhibit low surface energy and limited chemical reactivity, which can reduce irreversible protein denaturation and platelet interaction compared to more protein-retaining polymer matrices [15]. Experimental comparisons of polymers used in DES coatings have shown that hemocompatibility depends not only on total protein adsorption but also on the identity and persistence of adsorbed proteins, particularly the balance between albumin retention and fibrinogen-mediated platelet activation. Therefore, PVDF-HFP demonstrates a fundamental principle of cardiovascular coating design: a polymer can be clinically useful not only because it provides mechanical strength or controlled drug release, but also because it shapes the composition and biological activity of the interface protein layer [16].

In particular, acrylate and methacrylate carriers such as poly(n-butyl methacrylate) (PBMA) and poly(ethylene-co-vinyl acetate) (PEVA) have been used as liner/top coating and matrix layers in previous-generation devices (e.g., the PEVA/PBMA system of first-generation sirolimus-eluting stents). They form drug-permeable films, making them suitable for sustained release, but show higher protein retention and thrombogenicity compared to fluoropolymers in controlled comparisons [16]. A common disadvantage of persistent chemicals is persistence: a chronic foreign body stimulation associated with delayed endothelialization, hypersensitivity, and late thrombotic risk in early-stage devices [5].

### 2.2. Biodegradable Polyester Carriers

Biodegradable polyesters deliver the drug and are then absorbed, leaving no permanent implant on a bare platform (biodegradable polymer DESs) or fully biodegradable scaffolds [17]. Therefore, their degradation chemistry is inseparable from their biological behavior.

Poly(lactic-co-glycolic acid) (PLGA) degrades by bulk hydrolysis of ester bonds; the lactide–glycolide ratio and molecular weight regulate the degradation rate and, consequently, the drug release profile. Lactic and glycolic acids, which are hydrolysis products, can acidify the local microenvironment. Optimized PLGA-based and PLA/poly(ethylene-co-vinyl acetate) blend coatings are specifically designed to control film degradation and release behavior for next-generation biodegradable stents [18]. Poly(lactic acid)/poly(L-lactide) (PLA/PLLA) has a more crystalline structure and degrades more slowly, providing the structural polymer for various biodegradable scaffolds; poly(ε-caprolactone) (PCL) is a flexible, slow-hydrolyzing polyester that is often blended or copolymerized to fine-tune its mechanical and degradation properties. A characteristic disadvantage of this family is the delayed inflammatory wave accompanying bulk degradation, where acidic byproducts and particles act as danger signals. NET formation has been shown to contribute to the biocompatibility and degradation behavior of biodegradable scaffolds [11]. Abluminal and asymmetric designs that confine the biodegradable drug layer to the side facing the vessel represent a material-level response.

The biological challenge of biodegradable coatings is therefore both temporal and chemical. PLGA, PLA, and PCL do not offer a static interface: water uptake, ester hydrolysis, molecular weight reduction, pore formation, and mass loss progressively alter both drug delivery and the surface encountered by blood and tissue [19,20]. In PLGA-based coatings, degradation and erosion can directly correlate with drug release, producing release profiles different from those of purely diffusion-controlled, durable systems. These evolving material states are important for thrombo-inflammatory performance because the same processes that enable absorption can also produce acidic products, particulate residues, and altered surface roughness. Therefore, biodegradable coating design should aim not only to eliminate long-term polymer burden but also to synchronize degradation with endothelial healing and inflammation resolution [21].

### 2.3. Polymer-Free and Inorganic Contrast Strategies

To avoid polymer-related late events, polymer-free and inorganic coated platforms (microporous reservoirs, carbide or oxide surface layers) have been developed. These serve as an instructive comparison. When polymer removal reduces inflammation, it points to the carrier as the culprit component. Conversely, it highlights the functional value of the carrier when it disrupts release control [22]. Comparing polymer-containing and polymer-free strategies isolates the biological contribution of the coating material.

### 2.4. Hemocompatible and Bioactive Surface Chemistries

There is a distinct class of coatings designed to control the blood–material response rather than deliver an antiproliferative drug [23]. These chemistries are united by a single physical principle with strong and stable surface hydration that resists protein adsorption.

The phosphorylcholine (PC) chemistry and the methacrylate monomer 2-methacryloxyethyl phosphorylcholine (MPC) mimic the zwitterionic head group of the outer cell membrane. The resulting hydration layer and charge neutrality suppress protein and platelet adhesion. PC coatings were among the first biomimetic chemistries commercialized in coronary stents, and more recently, thin, covalently bonded MPC coatings have demonstrated strong antithrombogenicity in neurovascular stents without interfering with the endothelial coverage [24]. Poly(ethylene glycol) (PEG) grafting provides a hydrophilic, steric anti-fouling effect via a mobile hydrated brush, but oxidative susceptibility limits its long-term durability. Zwitterionic polymers, such as carboxybetaine, sulfobetaine, and MPC, form the most robust hydration layers and can provide virtually zero fouling. The ultra-low fouling behavior of zwitterionic materials is largely attributed to their tightly bound hydration shells, which form a significant energy barrier against non-specific protein adsorption [25]. Recent studies have addressed historical weaknesses such as mechanical stability and substrate adhesion by producing robust zwitterionic coatings and phosphocholine block copolymers with proven anticoagulant performance for catheters with complex geometries [26,27].

Among fouling prevention strategies, zwitterionic polymers are particularly important because their resistance to protein adsorption stems from a tightly bound hydration shell rather than simply passive hydrophilicity [28]. Carboxybetaine, sulfobetaine, and phosphorylcholine-based polymers possess paired positive and negative charges that organize water molecules at the interface and form an energetic barrier against non-specific protein adsorption. This mechanism distinguishes them from conventional hydrophilic coatings such as PEG, which rely primarily on steric repulsion and chain mobility and may be vulnerable to oxidative degradation over time. Several comparative studies have suggested that zwitterionic coatings may provide more durable antifouling performance than PEG-based systems under physiological conditions [29]. In devices that come into contact with blood, the practical value of zwitterionic chemistry lies in its ability to suppress protein adsorption, which represents the first step in the blood–material interaction cascade. By limiting protein adsorption, these coatings reduce platelet adhesion, leukocyte recruitment, and downstream thrombo-inflammatory activation [30,31].

Mussel-inspired polydopamine is a versatile anchoring chemistry rather than a passive carrier: catechol/quinone groups adhere to virtually any substrate and provide reactive sites for immobilizing heparin, peptides, or growth factors. For example, polydopamine coatings functionalized with vascular endothelial growth factor promote endothelial coverage while suppressing smooth muscle proliferation and neointimal formation in vivo [32]. Such bioactive surfaces are further developed as design strategies in Section 6.

### 2.5. Governing Surface Properties

**Figure 1:**
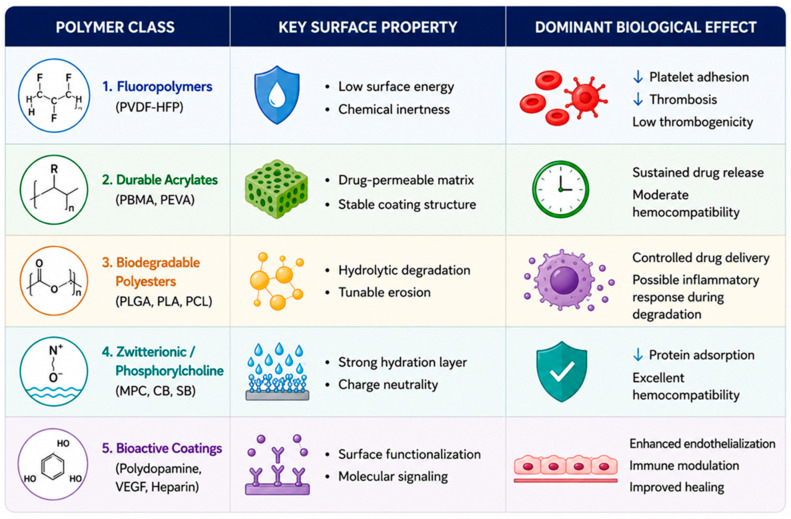
Polymer coating classes are matched with their defining surface properties and characteristic biological signatures. Durable fluoropolymers (e.g., PVDF-HFP) provide chemical inertness and reduced thrombogenicity, while durable acrylate systems primarily support sustained drug release. Biodegradable polyesters enable degradation-dependent drug delivery but may generate inflammatory degradation products. Phosphorylcholine and zwitterionic coatings create strongly hydrated anti-fouling surfaces that enhance hemocompatibility, while bioactive platforms such as polydopamine support endothelialization and tissue integration through molecular functionalization. The figure highlights the progression from material chemistry to surface properties and ultimately to biological performance.

In all these classes, the biological response is governed by a compact array of surface properties: wettability and hydrophilicity, surface charge and zeta potential, nanoscale roughness and topography, and the specific functional group chemistry presented to the blood. These parameters determine the composition and conformation of the adsorbed protein layer and, through this, downstream cell behavior [16,33,34]. Figure 1 summarizes the main classes of polymer coatings used in cardiovascular and endovascular devices and shows how their physicochemical properties translate into different biological signatures. Differences in surface hydration, hydrophobicity, degradation behavior, and bioactivity among coating platforms ultimately shape thrombogenicity, endothelialization, and inflammatory responses.

Building on this qualitative mapping, Table 1 consolidates representative quantitative values of principal coating polymers, along with representative clinical applications.

### 2.6. Cytotoxicity and Biocompatibility Assessment

Because coated devices reside in direct and prolonged contact with blood and vessel walls, the cytotoxicity and biocompatibility of the coating layer are as critical as mechanical or drug release performance. Biological safety is assessed according to the ISO 10993 series of standards for medical devices. In vitro cytotoxicity provides the initial screening of a candidate coating and its degradation products. In vitro cytotoxicity assessment is typically performed using ISO 10993-5, using extract, direct contact or indirect tests in standardized cell lines (e.g., L929 fibroblasts) with viability endpoints such as MTT or neutral red uptake [39]. For coatings in contact with blood, ISO 10993-4 specifies the selection of hemocompatibility tests in five domains, including thrombosis, coagulation, platelets, hematology and complement [40]. Hemolysis is quantified separately, most commonly by ASTM F756 [41].

These endpoints differ systematically across the coating classes summarized in Table 1. Durable fluoropolymers and phosphorylcholine/zwitterionic surfaces generally exhibit low cytotoxicity and low hemolytic and platelet-activating potential, consistent with their hydration and inertness-based heme compatibility. Biodegradable polyesters are cytocompatible in bulk but may cause transient local acidification during degradation. Therefore, cytotoxicity and inflammatory potential should be assessed not only on the manufactured coating but also on aged or degrading samples. Framing cytotoxicity and hemocompatibility as standardized, material-dependent endpoints, rather than a single pass/fail test, allows for the design and selection of coating chemistry for a defined biological safety profile.

## 3. Drug Release Kinetics as a Material-Governed Variable

In drug-containing coatings, release kinetics is not an independent property imposed on the device, but rather an emerging characteristic of the coating material, its architecture, and the application method. It is essential to treat release as a material phenomenon, as the profile determines not only the local drug concentration but also the timing and intensity of the inflammatory environment, as developed in Section 4 and Section 5.

### 3.1. Release Mechanisms

Drug release from a polymer coating occurs via three broad mechanisms, alone or in combination. Diffusion-controlled release is dominant in durable matrices where the drug migrates through the polymer and water-filled pore network at a rate determined by the diffusion rate and coating geometry. Degradation or erosion-controlled release is dominant in biodegradable polyesters where hydrolytic chain breaking gradually increases permeability and porosity, and release accelerates as the matrix erodes. Reservoir (membrane-controlled) and matrix (monolithic) architectures represent another design axis. The first provides near-zero-order release via a rate-limiting top coating, while the second offers a decreasing profile. In practice, most coatings are hybrid in structure: for example, sirolimus release from PLGA is governed by diffusion and degradation, and the loading level has relatively little effect on the shape of the profile [42,43].

### 3.2. Material Levers on Release Profile

Since release is governed by the material, designers tune it with a defined set of effects: polymer chemistry and molecular weight (determining the rate of diffusion and degradation), lactide–glycolide ratio in PLGA, coating thickness, drug-polymer ratio, crystallinity, and multilayer architecture. Modeling and experiments agree that the profile of a biodurable everolimus coating is controlled by characteristic parameters such as coating thickness and diffusion coefficient [44]. An ongoing practical challenge is burst release, in which a substantial fraction of the drug is released immediately after implantation. This phenomenon can reduce delivery efficiency and increase the risk of local toxicity. This effect can be suppressed with multilayer or top coating designs and abluminal/asymmetric coating that limits and slows the release. Asymmetric biodegradable coatings show slower, more stable, less abrupt release than conventional symmetric coatings both in vitro and in vivo [42].

### 3.3. Manufacturing Method as a Determinant Factor of Release

The coating technique is a release variable in itself as it determines film homogeneity, thickness control, and drug-polymer microstructure. Dip coating, spray coating, ultrasonic atomization, electrospinning, and electrostatic dry powder deposition methods each produce characteristic morphologies and repeatability, and the resulting differences in surface roughness and layer integrity translate into different release profiles and ultimately different biological responses [18,42]. This connects Section 3 back to the surface feature arguments in Section 2: how a coating is made partly determines the surface that blood and tissue encounter.

### 3.4. Drug-Coating Pairing and the Drug-Coated Balloon Paradigm

Different therapeutic agents present different release requirements. Limus family drugs (sirolimus, everolimus, zotarolimus) are typically lipophilic mTOR inhibitors formulated for sustained release from a retained film, whereas paclitaxel is generally used for rapid transfer. The drug-coated balloon (DCB) represents the extreme end: there is no permanent scaffold, and the entire therapeutic dose must be transferred to and retained in the vessel wall during a brief inflation [36]. Therefore, the material challenge shifts from sustained release to efficient, durable tissue uptake, which largely depends on the drug’s excipient and crystalline form. Clinical heterogeneity among DCB devices reflects this sensitivity [7,37,45].

### 3.5. Modeling and Link to Local Biology

Mechanistic transport and erosion models linking polymer degradation to drug diffusion have been developed and validated with experimental data. These models clearly demonstrate how molecular weight reduction, porosity evolution, and drug distribution profile collectively determine release kinetics and local drug availability [44]. Importantly, release is also modulated by the in vivo environment: fluid shear stress modifies the in vitro release kinetics of sirolimus from PLGA films, creating a multiphase profile; this highlights that the local flow field (Section 5.1) provides feedback to the release behavior that the coating is designed to produce [46]. These are materials science tools; computational and machine learning approaches to coating and device optimization have been discussed separately elsewhere [12]. The conceptual linkage carried forward is that the release profile determines the temporal drug and inflammation environment at the interface and links this section to both hemocompatibility and thrombo-inflammatory processes that follow.

### 3.6. From Release Kinetics to Immune Effect

Release kinetics are not only a pharmacological variable but also directly shape the local immune and inflammatory environment, providing a mechanistic bridge to the thrombo-inflammatory responses developed in Section 5. Three links are well supported. First, a large initial burst delivers a supratherapeutic local dose that can be cytotoxic to endothelial and smooth muscle cells and provoke acute inflammation, whereas controlled or sustained profiles limit this insult [28]. Second, sustained antiproliferative elution (limus and paclitaxel agents) suppresses neointimal proliferation while simultaneously delaying endothelial healing and prolonging exposure to a thrombogenic, incompletely healed surface, which is a key element of the restenosis-healing balance [5,6]. Third, in biodegradable carriers, the kinetics of degradation govern the timing and magnitude of acidic byproduct release. These acts as a danger signal promoting macrophage activation and NET formation [11]. Therefore, the same material parameters that define the release profile also determine the rate and intensity of the immune response, as summarized in Table 2.

## 4. The Coating–Blood Interface: Protein Adsorption and Hemocompatibility

Within seconds of contact with blood, a polymer surface acquires a layer of adsorbed plasma proteins, and it is this layer, rather than the bare polymer, that cells subsequently encounter. Adsorbed proteins act as primary mediators of subsequent cellular interactions with biomaterial surfaces and therefore play a central role in determining biocompatibility outcomes [47]. The composition of the adsorbed layer evolves over time via competitive, surface-bound exchange through the classic Vroman effect [48]. In this effect, initially abundant, mobile proteins are gradually replaced by higher-affinity species [33]. Surface chemistry, charge, and hydrophobicity determine which proteins are dominant and whether they retain their native conformations. For example, adsorption-induced unfolding of fibrinogen reveals binding motifs that support platelet adhesion and activation.

The adsorbed proteome determines the course of three interlocking systems. When surface-bound factor XII and related zymogens are converted into active enzymes, contact and coagulation activation can be triggered, initiating the intrinsic pathway. Tissue factor exposure feeds the extrinsic pathway. Complement activation proceeds in a surface-dependent manner and amplifies inflammatory signaling. Platelet adhesion and activation, largely driven by adsorbed fibrinogen and von Willebrand factor, provide a coagulation-promoting surface and attract leukocytes. Hydrophilic, strongly hydrated, and charge-neutral surfaces (especially zwitterionic and phosphorylcholine chemistries) suppress this cascade primarily by limiting protein adsorption [26,27,49].

**Figure 2:**
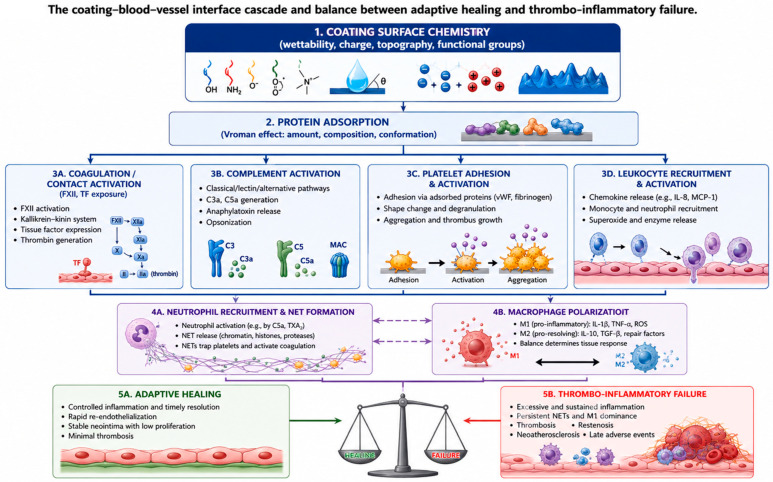
Coating–blood vessel interface cascade and the balance between adaptive recovery and thrombo-inflammatory failure. Surface chemistry, including wettability, charge, topography, and functional group presentation, governs the composition and conformation of the adsorbed protein layer. This interface then regulates coagulation and complement activation, platelet adhesion, leukocyte recruitment, neutrophil extracellular trap (NET) formation, and macrophage polarization. The cumulative balance between these responses determines whether the implanted device will progress toward adaptive recovery, characterized by rapid endothelialization and stable integration, or toward thrombo-inflammatory failure, characterized by thrombosis, restenosis, neoatherosclerosis, and adverse vascular remodeling.

Therefore, hemocompatibility is best understood as a downstream output of the coating surface chemistry and assessed through corresponding standardized domains (thrombosis, coagulation, platelet activation, hematology, and complement] in the cascade described above. Framing these endpoints not as independent pass/fail tests but as outcomes of material parameters allows for the design of coating chemistry for a targeted blood response. As shown in Figure 2, the biological response to a coated device begins within seconds of contact with blood and develops through a coordinated network of coagulation, complement, platelet, neutrophil, and macrophage pathways. The balance between these processes determines whether the implanted surface will progress toward adaptive healing or sustained thrombo-inflammatory activation. Understanding this interface step provides the mechanistic basis for the outcome-oriented discussion presented in Section 5. This concept aligns with the broader view that biocompatibility is not an intrinsic property of the material but the result of dynamic host–material interactions [50].

## 5. Thrombo-Inflammatory Response to Coating Materials

The hemocompatibility cascade does not act in isolation. It is the entry point for a broader thrombo-inflammatory response that determines whether an implanted, coated device will recover or fail. This response is the central determinant of clinical outcome and is where the defining effects of coating material parameters are demonstrated.

### 5.1. Immunothrombosis on Coated Surface

Vascular injury and foreign body contact combine coagulation with innate immunity in a process called immunothrombosis. Platelets adhering to the adsorbed protein layer release P-selectin and release chemokines that recruit and activate leukocytes. The activated leukocytes then release tissue factor and provide coagulation-supporting surfaces. Therefore, hemostasis and inflammation reinforce each other. Under physiological control, this supports sealing and repair, but when it becomes disorganized, it leads to occlusive thrombus formation and maladaptive remodeling [9,10]. The coating material influences this process from the earliest stage of blood–material interaction, as the surface chemistry governing protein adsorption (Section 4) also determines the initial platelet and leukocyte activation state.

Local hemodynamics are an integral codeterminant. Stent struts and surface irregularities create low and oscillatory shear zones that promote endothelial activation, leukocyte adhesion, and a pro-inflammatory, pro-thrombotic endothelial phenotype, while high laminar shear is protective [51,52]. Clinically, neointimal thickness on struts is inversely proportional to local endothelial shear stress and is associated with incomplete strut placement, delayed sheathing, and late thrombosis, which increase flow disturbance [4]. Therefore, coating thickness, hemocompatibility, and strut profile influence biology, partly through their effects on the local shear zone. This is another way in which material selection becomes a biological choice.

### 5.2. Neutrophils and Neutrophil Extracellular Traps

Neutrophils are the earliest leukocytes to arrive at an injured or foreign surface, and NETs, which are decondensed chromatin networks coated with histones, neutrophil elastase, and myeloperoxidase, have undergone a transformation from a purely antimicrobial role to recognition as central effectors of cardiovascular thrombosis and inflammation [53,54,55]. Increasing evidence also indicates that NETs are associated with sterile inflammatory states, thrombosis, and biomaterial-related immune responses [56,57]. Mechanistically, NETs provide a coagulation-supporting backbone: the DNA-histone backbone binds and activates platelets, histones are directly cytotoxic and prothrombotic, and bound enzymes enhance both intrinsic and extrinsic coagulation, while von Willebrand factor protects platelet chains from degradation [58].

This biology is directly relevant to coated devices. NET components are enriched in extracted coronary thrombi, and coronary NET load predicts infarct size and outcome in acute coronary syndrome [38,59,60]. Device-induced NETosis is increasingly documented in blood-contact systems, as in the example of extracorporeal membrane oxygenation where circulating NET markers are associated with coagulation disorders [61]. NET formation is also mechanosensitive; so, the disrupted flow microenvironment created by struts can itself promote NETosis. Critical to this investigation is how the chemistry, topography, and degradation products of the biomaterial surface modulate the neutrophil response, and NET formation has been shown to shape the biocompatibility and degradation behavior of bioabsorbable vascular scaffolds [11,62]. This principle generalizes across coating classes: the chemistry and degradation profile presented to the recruited neutrophils helps determine the magnitude of the NET response and thus the thrombotic scaffold it forms.

### 5.3. Macrophages and Foreign Body Response

Beyond the acute neutrophil phase, monocytes and macrophages dominate the subacute and chronic response and largely determine its resolution [11,49]. Depending on the adsorbed protein coat and the physical and chemical cues of the surface they encounter, macrophages adopt a range of phenotypes encompassing pro-inflammatory (M1-like) and reparative (M2-like) states. The importance of macrophage phenotype in determining biomaterial integration is widely recognized in regenerative medicine and implantable device research [63,64,65]. Failure of the transition from M1 to M2 sustains inflammation, foreign body giant cell formation, and fibrotic or neointimal overgrowth [34,66]. Importantly, cues influencing this balance, such as surface chemistry and functional groups, wettability, nanoscale topography and roughness, and substrate stiffness, are all coating design variables. This makes the foreign body response a modifiable target rather than a fixed liability and provides the mechanistic basis for the immunomodulatory surfaces discussed in Section 6.

### 5.4. Degradation Products and Polymer Hypersensitivity

Biodegradable coatings add a temporal dimension to inflammation. Acidic hydrolysis products and particulate residues from PLGA/PLA can lower the local pH and act as danger signals, creating a delayed inflammatory wave during bulk degradation that distinguishes absorbable systems from resistant systems [11,17]. In contrast, resistant fluoropolymers have been associated with hypersensitivity reactions linked to delayed events in some cases. In both cases, the inflammatory stimulus is a direct characteristic of the coating material and degradation chemistry.

### 5.5. Resolution Aspect: Endothelialization

The desirable resolution of thrombo-inflammatory activation is rapid and complete re-endothelialization, which restores a non-thrombogenic, anticoagulant luminal surface and limits intimal overgrowth. The incomplete endothelial coverage of stent struts is a major cause of late-stage stent thrombosis [5,6]. Coating chemistry greatly influences this outcome and creates the defining tension of the field: antiproliferative drugs that suppress smooth muscle proliferation to prevent restenosis also delay endothelial healing; so, a coating adjusted solely for restenosis control may disrupt healing. Pro-endothelial and biomimetic surfaces (Section 6) aim to separate these effects by allowing for or actively promoting endothelial coverage while inhibiting smooth muscle proliferation. Thus, the restenosis-healing balance is ultimately determined at the coating surface. Taken together, these findings demonstrate that coating chemistry and surface properties influence clinical outcomes beyond the pharmacological effects of the applied drug.

### 5.6. A Cohesive Structure–Response Map

These mechanisms, taken together, support a single regulatory claim: not all polymer coatings are biologically equivalent. Surface chemistries and degradation profiles shape the thrombo-inflammatory responses that ultimately determine healing, restenosis, and thrombosis. While Table 1 (Section 2) summarizes the material properties of each coating classes, Table 3 illustrates the broader structure–property–biological response relationships governing thrombo-inflammatory performance. These pairings highlight that biological outcomes stem not solely from coating category but from specific surface properties presented to blood and tissue.

Representative examples illustrating how coating properties influence interface biology and, consequently, clinical performance. The relationships shown are conceptual and may vary depending on device type, local hemodynamics, drug load, and patient-specific factors.

## 6. Material Design Strategies to Improve Thrombo-Inflammatory Performance

**Figure 3:**
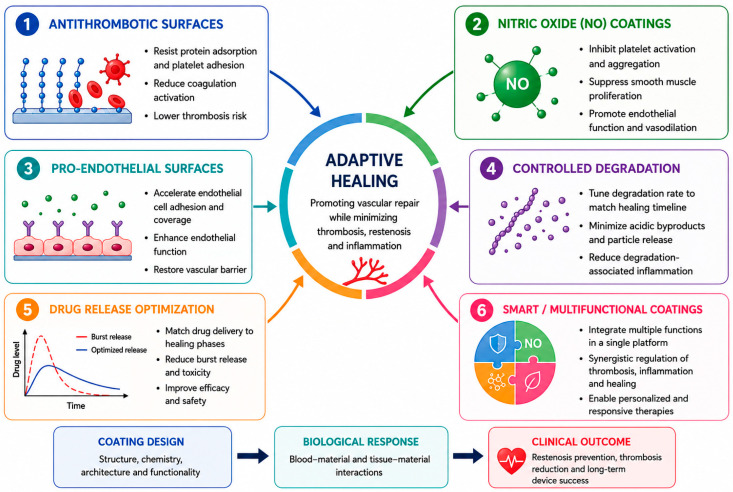
Material design strategies for modulating thrombo-inflammatory responses in cardiovascular and endovascular polymer coatings. Contemporary coating platforms increasingly integrate multiple complementary approaches, including antithrombotic surface engineering, nitric oxide-releasing or nitric oxide-producing systems, pro-endothelial and biomimetic interfaces, controlled degradation strategies, optimized drug release kinetics, and multifunctional smart coatings. The common goal is to shift the biological response toward adaptive recovery while minimizing thrombosis, restenosis, chronic inflammation, and late device failure. The central framework emphasizes coating design as a structure–property–response paradigm in which material parameters are selected based on their intended biological effects.

If coating parameters determine the biological response, then improving device outcomes essentially becomes a material design problem [67,68], as summarized in Figure 3. The following strategies are arranged according to the point they target in the stepwise process in Section 5: suppressing adverse blood response, providing protective signaling molecules, accelerating healing, controlling degradation, and actively directing the immune response. In practice, these approaches are increasingly combined, as no single mechanism can resolve the restenosis-healing balance alone.

Figure 3 provides a framework for understanding contemporary material design strategies aimed at shifting the coating–host interaction towards adaptive healing. These approaches target multiple components of the thrombo-inflammatory process, including protein adsorption, platelet activation, endothelial healing, degradation-related inflammation, and immune regulation, rather than focusing solely on drug delivery.

### 6.1. Antithrombotic Surface Engineering

The most direct approach is to minimize protein and platelet adhesion on the surface by attacking the cascade in the first stage. Heparinized surfaces offer inert anticoagulant activity at the interface, whereas biomimetic hydration-based chemistries resist initial protein adsorption that triggers activation. Phosphorylcholine and zwitterionic polymers (carboxybetaine, sulfobetaine, and MPC) form a strongly bonded, charge-neutral hydration layer that provides very low fouling. For example, covalently attached MPC coatings significantly reduce thrombus formation in neurovascular stents without obstructing the endothelial covering [24]. Long-standing hurdles have been mechanical durability and adhesion to the substrate under pulsatile loading, and recent chemistries directly target these: durable zwitterionic coatings have been demonstrated on long and complex geometry catheters, and phosphocholine block copolymers with confirmed anticoagulant performance and in vivo safety have been reported [26,27].

### 6.2. Nitric-Oxide-Releasing and Generating Coatings

A second strategy provides a signaling molecule that protects the surface rather than simply passivating it. Nitric oxide (NO) is an endogenous inhibitor of platelet activation and smooth muscle proliferation and a promoter of endothelial health [69,70]. Therefore, a NO-supplying coating could in principle promote healing while suppressing thrombosis and restenosis, thereby addressing all three components of the healing–restenosis–thrombosis balance. Two material approaches are available: NO-releasing coatings that store and release donor species, and NO-generating coatings that carry catalysts (e.g., copper- or selenium-based sites) that generate NO from endogenous S-nitrosothiols. The key material challenge is to maintain a physiologically relevant flow of NO for a clinically useful period. This has led to tethered donor chemistries and catalytic surfaces designed to prolong and localize NO production [71]. Localized NO delivery has been extensively investigated as a strategy to both improve hemocompatibility and reduce inflammatory activation at biomaterial interfaces [58].

### 6.3. Pro-Endothelial and Biomimetic Surfaces

A third strategy directly accelerates the positive healing pathway. Endothelial progenitor cell capture surfaces, endothelium-mimicking coatings, and biomimetic extracellular matrix layers aim to regenerate a unified, non-thrombogenic endothelium more rapidly and shift the restenosis-healing equilibrium towards resolution [62]. Mussel-inspired polydopamine is very useful here and provides an almost universal anchoring layer for immobilizing endothelium-selective signals: polydopamine surfaces functionalized with vascular endothelial growth factor suppress smooth muscle proliferation and neointimal formation in vivo while promoting endothelial coverage [32]. Because these surfaces inhibit the proliferation of smooth muscle cells while allowing for the growth of endothelial cells, they begin to separate the two effects brought together by an antiproliferative drug.

Clinically defined coating and support modifications measurably narrow the antiproliferative-healing balance. Reducing support thickness and adopting bioabsorbable polymer carriers accelerates support endothelialization and reduces vascular damage and inflammation. In the randomized BIOFLOW V study, ultra-thin support (60 µm) bioabsorbable polymer sirolimus-releasing stents produced significantly lower rates of three-year target lesion failure and target vessel myocardial infarction, clinically guided target lesion revascularization, and late/very late stent thrombosis compared to thin-support (81 µm) durable polymer everolimus-releasing stents [72]. This demonstrates that material-level modifications (thinner, more compliant, absorbable coatings) improve clinical healing without compromising restenosis control.

### 6.4. Tuning Degradation to Flatten the Inflammatory Wave

The design goal for biodegradable systems is the temporal inflammatory profile itself. The degradation behavior of biodegradable coatings is governed by factors such as lactide–glycolide ratio, molecular weight, crystallinity, coating thickness, and layer architecture. Together, these parameters determine the rate of formation of acidic degradation products and particulate residues, thereby influencing the magnitude of the delayed inflammatory response associated with bulk polymer degradation [17,18]. Matching the degradation timeline to the vessel healing timeline, so that the coating disappears as the endothelium repairs, is a clear design goal in itself, and the NET-modulating behavior of degradable scaffolds is a directly related issue [11].

### 6.5. Immunomodulatory and Bioactive Coatings

The latest frontier is moving from suppression or support to actively directing the host response. Immunomodulatory coatings are designed to direct macrophage polarization toward a reparative M2-like phenotype through defined surface chemistry, topography, and stiffness cues, transforming the foreign body response from a disadvantage into a controllable variable [34,62]. Extending this logic, extracellular vesicle-inspired and other biologically active interfaces aim to deliver naturally, like signaling on the surface. These orientations, along with the broader concept of immunologically informed and adaptive device design, have been developed in detail elsewhere [49] and are presented here as the natural endpoint of the structure–property–response framework developed throughout this review. The key point for coating designers is that the immune response is now a design target that can be addressed through material parameters. Collectively, these strategies demonstrate a continuing transition from passive drug carriers to biologically directive interfaces that can actively regulate thrombosis, inflammation, and healing.

Increasing evidence shows that a single coating strategy cannot simultaneously optimize thrombosis prevention, endothelial repair, drug delivery, and immune regulation. Consequently, contemporary device development is moving towards multifunctional coating architectures that integrate complementary biological mechanisms on a single platform. This transition reflects a broader trend in biomaterial engineering toward multifunctional interfaces that can simultaneously modulate thrombosis, inflammation, and tissue repair [51]. Examples include coatings that combine antiproliferative drug release with nitric oxide production, zwitterionic anti-fouling layers with endothelial-supporting ligands, or biodegradable matrices containing immunomodulatory molecules. Such systems aim to address the limitations of conventional single-function coatings by simultaneously targeting multiple phases of the thrombo-inflammatory cascade. Therefore, it is likely that future cardiovascular coatings will be evaluated not only according to their drug release performance but also according to their capacity to regulate coordinated biological responses throughout thrombosis, inflammation, and vascular repair [55,65].

Table 4 summarizes the major emerging coating approaches, their dominant biological targets, and expected clinical benefits.

Contemporary cardiovascular and endovascular coatings are increasingly moving from passive drug delivery to active biological regulation. The table summarizes the major design approaches aimed at modulating thrombosis, inflammation, endothelial healing, and long-term device integration. Many next-generation platforms combine multiple strategies within multifunctional coating architectures.

## 7. Translation Challenges, Standardization, and Future Directions

Several shortcomings limit the transfer of coating design from the lab to the clinic. First, tests are not standardized: both compatibility tests capture coagulation, platelet, and complement endpoints, but there are no widely accepted protocols to measure NET formation or macrophage polarization on candidate coatings under physiological flow, making inter-study comparison difficult [34,55]. Second, structure–feature–response datasets that systematically link defined coating chemistries to immune endpoints are still scarce; so, design still relies heavily on empirical iteration. Third, the gaps between in vitro–in vivo and preclinical–clinical are wide, as static or short-term tests inadequately reproduce the evolving, flow-dependent biology of a healing vessel. One of the biggest obstacles to rational coating development is the lack of compatible biological evaluation frameworks.

Closing the gap between in vitro and in vivo outcomes requires established standards implemented through a phased testing strategy. The first phase is standardized in vitro screening. Cytotoxicity can be assessed under ISO 10993-5 and hemocompatibility under ISO 10993-4, while hemolysis is measured by ASTM F756 [39,40,41]. The second phase adds flow-based functional assays that reproduce physiological shear and measure thrombus formation, platelet adhesion and leukocyte adhesion, and ideally NET and macrophage responses. Harmonized protocols for these assays are still lacking. The third phase is in vivo correlation in appropriate animal models using predefined histopathological and endothelialization endpoints. A hurdle persists throughout the entire process. Static or short-term in vitro assays do not reproduce the evolving, flow-dependent biology of a healing vessel. Better alignment between standardized regulatory endpoints and dynamic, immune-sensitive assays will improve how reliably coating assessment predicts clinical performance.

Table 5 summarizes representative experimental endpoints that can be used to characterize coating performance along the thrombosis–inflammation–healing continuum.

The table highlights the progression from early interface events (protein adsorption and platelet activation) to downstream thrombo-inflammatory responses, vascular healing, and long-term device performance. Standardized assessment in these areas can improve comparison between studies and facilitate the implementation of next-generation coating technologies.

Closing these gaps will require standardized flow-based immunoprofiling of coatings (NET, macrophage, and endothelial readouts), compatible biomarker panels, and prospective correlation of coating properties with clinical outcomes. Adjacent questions such as AI-driven design and immune-adaptive device frameworks are not covered here and are addressed separately [12,49]. The priority in the materials domain is to make coating chemistry determinable according to the intended biological response. The future is a shift from coatings primarily defined by their drug release profile to those defined by the thrombo-inflammatory and healing response they are designed to produce. Beyond traditional compatibility testing, future evaluation frameworks may increasingly include thrombo-inflammatory biomarkers, endothelial recovery measures, and longitudinal assessments of degradation-related immune responses. Integration of these endpoints into standardized preclinical testing processes can facilitate comparison between coating platforms and accelerate the translation of next-generation cardiovascular biomaterials. These approaches may be particularly important for multifunctional and bioactive coatings whose biological performance extends beyond traditional drug release endpoints.

## 8. Conclusions

Polymer coatings are the defining interface of cardiovascular and endovascular devices and are not biologically inert. Their surface chemistry, drug release kinetics, and degradation behavior govern protein adsorption, biocompatibility, and thrombo-inflammatory responses (including NET formation, macrophage polarization, and endothelial recovery), which ultimately influence restenosis and thrombosis. Reconsidering the coating development process as a structure–property–biological response problem reveals both the limitations of traditional drug-centric design and a clear path forward. Future coatings should be designed, characterized, and selected according to the biological responses they are intended to generate. Consequently, clinical performance is determined not only by material composition but also by the biological responses generated by the coatings at the device–tissue interface.

## Figures and Tables

**Table 1:** The physicochemical and biological properties of the principal polymer coating classes used in cardiovascular diseases are presented, along with representative clinical applications. Values are representative ranges from the literature and vary depending on quality, molecular weight, and processing.

Polymer (Class)	Thermal (Tg/Tm)	Mechanical	Wettability (Water CA)	Degradation	Biocompatibility	Representative Clinical Application	References
PVDF-HFP (durable fluoropolymer)	Tg ≈ −35 to −20 °C; semi-crystalline	Flexible, durable film	Hydrophobic (≈90–100°)	Non-degradable	Inert; low thrombogenicity (fluoropassivation)	Durable polymer everolimus-eluting stents	[6,16]
PBMA/PEVA (durable acrylate)	Tg ≈ 20–35 °C	Soft, film-forming	Moderately hydrophobic (≈70–85°)	Non-degradable	Biocompatible; higher protein retention/thrombogenicity than fluoropolymer	First-generation sirolimus- eluting stent; primer/topcoat layers	[16]
PLGA (biodegradable polyester)	Tg ≈ 40–55 °C; amorphous	Rigid, brittle	Moderately hydrophobic (≈70–80°)	Weeks–months (ratio dependent: 50:50 ≈1–2 months)	Biocompatible; acidic degradation products	Biodegradable polymer DESs	[28,35]
PLLA/PLA (biodegradable polyester)	Tg ≈ 55–65 °C; Tm ≈ 170–180 °C	Stiff, semi-crystalline	Hydrophobic (≈80°)	Months–years	Biocompatible; scaffold material	Bioresorbable scaffolds; ultra-thin BP-SES coatings	[28,35]
PCL (biodegradable polyester)	Tg ≈ −60 °C; Tm ≈ 55–60 °C	Ductile, flexible	Hydrophobic (≈80°)	1–2+ years (slow)	Biocompatible	Slow-release depots and polymer blends (limited standalone stent use)	[35]
Phosphorylcholine/zwitterionic	Amorphous; hydrogel-like	Soft, hydrated layer	Superhydrophilic (<40°)	Non-degradable (durable)	Excellent hemocompatibility; low protein/platelet adhesion	Phosphorylcholine-coated stents; catheter coatings	[36,37]
(MPC) Polydopamine (bioactive anchor)	Thin adherent film	Robust anchoring layer	Hydrophilic (≈40–60°)	Stable	Biocompatible, bioactive; supports endothelialization	Bioactive surface functionalization (under investigation; EPC capture, growth factor immobilization)	[38]

**Table 2:** Direct evidence chain linking drug release behavior to immune/inflammatory effects.

Release Feature	Material Origin	Immune/Inflammatory Effect	Consequence
Initial burst	Thin/loaded matrix; no topcoat	Local cytotoxicity; acute inflammation	Endothelial/SMC injury; impaired healing [28]
Sustained antiproliferative elution	Durable film; limus/paclitaxel	Delayed re-endothelialization	Prolonged thrombogenic surface; late thrombosis [5,6]
Degradation-coupled acid release	PLGA/PLA hydrolysis	Macrophage activation; NET formation	Second inflammatory wave; scaffold phase thrombosis [11]

**Table 3:** Structure–property–biological response relationships governing the thrombo-inflammatory performance of cardiovascular polymer coatings.

Material Property	Interface Effect	Biological Response	Clinical Consequence
High hydration (MPC, zwitterionic)	Reduced protein adsorption	Reduced platelet activation	Lower thrombosis risk
High hydrophobicity	Fibrinogen unfolding	Increased platelet adhesion	Higher thrombogenicity
Acidic decomposition products	Reduction in local pH	Macrophage activation, NET formation	Delayed healing
Rough topography	Changes in protein layer and shear microenvironment	Enhanced leukocyte recruitment	Variable thrombogenicity
High antiproliferative exposure	Suppressed SMC proliferation	Delayed endothelialization	Reduced restenosis but increased healing delay
Pro-endothelial signaling	Accelerated endothelial coverage	Restored vascular barrier	Lower late thrombosis risk

**Table 4:** Emerging polymer coating strategies, primary biological targets, and expected clinical benefits.

Strategy	Mechanism	Primary Biological Target	Potential Clinical Benefit	Development Status
Zwitterionic coatings	Hydration-mediated anti-fouling through strong bound water layers	Protein adsorption, platelet activation	Reduced thrombosis and improved hemocompatibility	Clinical/commercial use emerging
Nitric oxide (NO) generating coatings	Catalytic conversion of endogenous NO donors at the surface	Platelets, SMCs, endothelium	Simultaneous reduction in thrombosis and restenosis	Advanced preclinical to early clinical
Nitric oxide (NO) releasing coatings	Controlled release of stored NO donor species	Platelets, SMCs, endothelium	Enhanced vascular healing and thromboresistance	Preclinical
Heparinized coatings	Surface-bound anticoagulant activity	Coagulation cascade, thrombin formation	Improved hemocompatibility and reduced acute thrombosis	Clinical use on selected devices
Pro-endothelial coverages	EPC capture, VEGF immobilization, endothelial signaling	Endothelial repair and coverage	Accelerated endothelialization and reduced late-stage thrombosis	Preclinical to early clinical
Biomimetic extracellular matrix (ECM) coatings	Presentation of native adhesive peptides and matrix cues	Endothelial and immune cell interactions	Improved integration and increased physiological healing	Experimental/preclinical
Immunomodulatory coatings	Surface-driven M2 macrophage polarization and inflammation control	Foreign body response, chronic inflammation	Reduced inflammatory remodeling and restenosis	Emerging preclinical
EV-inspired coatings	Biomimetic signaling using extracellular vesicle-inspired interfaces	Endothelium, macrophages, and immune pathways	Enhanced integration and adaptive healing	Experimental
Controlled degradation coatings	Tuned polymer chemistry and degradation kinetics	Degradation-associated inflammation	Reduced delayed inflammatory response	Clinical and preclinical
Smart response coatings	Triggered drug release in response to pH, ROS, enzymes, or flow	Multiple thrombo-inflammatory pathways	Personalized and adaptive biological control	Experimental
Multifunctional hybrid coatings	Integration of antithrombotic, pro-endothelial, and immunomodulatory elements in a single platform	Multiple pathways simultaneously	Simultaneous control of thrombosis, inflammation, and healing	Emerging next-generation platforms

**Table 5:** Representative biological endpoints, experimental tests, and translational significance for the evaluation of cardiovascular polymer coatings.

Biological Endpoint	Typical Assay(s)	Biological Significance	Relevance to Device Failure	Translational Importance
Protein adsorption	ELISA, SPR, QCM-D, mass spectrometry	Initial biomaterial–blood interaction	Triggers thrombosis and inflammation	Fundamental screening endpoint
Platelet adhesion and activation	SEM, flow cytometry, P-selectin expression, platelet aggregation assays	Early thrombogenic response	Acute and subacute thrombosis	Critical for blood-contact devices
Coagulation activation	Thrombin formation, TAT complexes, FXII activation assays	Activation of intrinsic and common coagulation pathways	Device thrombosis	Core biocompatibility measure
Complement activation	C3a, C5a, SC5b-9 quantification	Innate immune activation	Chronic inflammation and adverse remodeling	Increasingly recognized regulatory endpoint
Leukocyte recruitment	Flow adhesion assays, immunostaining, cytokine profiling	Early inflammatory response	Persistent vascular inflammation	Useful indicator of interface biocompatibility
NET formation	MPO-DNA complexes, citrullinated histone H3 (CitH3), immunofluorescence microscopy	Immunothrombosis and thrombo-inflammatory amplification	Thrombosis, restenosis, scaffold degradation	Emerging high-value biomarker
Macrophage polarization	CD86/CD206 markers, cytokine profiling, gene expression analysis	Foreign body response and tissue remodeling	Chronic inflammation and neointimal hyperplasia	Important for long-term integration
Endothelialization	CD31, VE-cadherin staining, endothelial coverage assays	Restoration of vascular barrier function	Delayed healing and late thrombosis	Major determinant of device success
Smooth muscle cell proliferation	BrdU incorporation, Ki-67 staining, proliferation assays	Neointimal formation and restenosis	Luminal narrowing	Key efficacy endpoint for DES/DCB platforms
Oxidative stress response	ROS tests, oxidative biomarker analysis	Cellular damage and inflammatory signaling	Chronic vascular dysfunction	Relevant for degradable and drug-eluting systems
Degradation-associated response	Mass loss studies, pH monitoring, degradation product characterization	Local tissue response to polymer degradation	Delayed inflammation and scaffolding failure	Essential for biodegradable coatings
In vivo vascular healing	Histology, OCT, IVUS, micro-CT, animal implantation studies	Integrated assessment of thrombosis, inflammation, and repair	Overall device performance	Highest translational relevance

## Data Availability

The datasets supporting the findings of this review are cited within this article and are available in the referenced original publications.
